# Supplementary material for: A Critical View of the Application of the APEX Software (Aqueous Photochemistry of Environmentally-Occurring Xenobiotics) to Predict Photoreaction Kinetics in Surface Freshwaters
Source: Molecules. 2019 Dec 18;25(1):9. doi: 10.3390/molecules25010009 (PMC7017383; doi:10.3390/molecules25010009)
Supplement: Supplementary file 1 [file molecules-25-00009-s001.zip › 3.2.4_gcc-4.4.0.zip!/3.2.4_gcc-4.4.0/3.2.4_gcc-4.4.0/doc/octave/html/interpreter/Searching-Properties.html]

Searching Properties - Untitled


Previous: Surface Properties,
Up: Graphics Object Properties


---

##### 15.2.2.9 Searching Properties

— Function File: h = **findobj** ()  
— Function File: h = **findobj** (prop\_name, prop\_value)  
— Function File: h = **findobj** ('-property', prop\_name)  
— Function File: h = **findobj** ('-regexp', prop\_name, pattern)  
— Function File: h = **findobj** ('flat', ...)  
— Function File: h = **findobj** (h, ...)  
— Function File: h = **findobj** (h, '-depth', d, ...)  
> Find object with specified property values. The simplest form is
>
> ```
>           findobj (prop_name, prop_Value)
> ```
>
> which returns all of the handles to the objects with the name
> prop\_name and the name prop\_Value. The search can be limited
> to a particular object or set of objects and their descendants by
> passing a handle or set of handles h as the first argument to
> `findobj`.
>
> The depth of hierarchy of objects to which to search to can be limited
> with the '-depth' argument. To limit the number depth of the hierarchy
> to search to d generations of children, and example is
>
> ```
>           findobj (h, '-depth', d, prop_Name, prop_Value)
> ```
>
> Specifying a depth d of 0, limits the search to the set of object
> passed in h. A depth d of 0 is equivalent to the '-flat'
> argument.
>
> A specified logical operator may be applied to the pairs of prop\_Name
> and prop\_Value. The supported logical operators are '-and', '-or',
> '-xor', '-not'.
>
> The objects may also be matched by comparing a regular expression to the
> property values, where property values that match `regexp
> (`prop\_Value`,` pattern`)` are returned. Finally, objects may be
> matched by property name only, using the '-property' option.
>
>
>
> **See also:** get, set.

— Function File: h = **findall** ()  
— Function File: h = **findall** (prop\_name, prop\_value)  
— Function File: h = **findall** (h, ...)  
— Function File: h = **findall** (h, "-depth", d, ...)  
> Find object with specified property values including hidden handles.
>
> This function performs the same function as `findobj`, but it
> includes hidden objects in its search. For full documentation, see
> `findobj`.
>
>
>
> **See also:** get, set, findobj, allchild.
